# Circulatory dietary and gut-derived metabolites predict early cognitive decline

**DOI:** 10.1080/19490976.2026.2649487

**Published:** 2026-03-27

**Authors:** Emily Connell, Saber Sami, Mizanur Khondoker, Anne Marie Minihane, Matthew G. Pontifex, Michael Müller, Simon McArthur, Gwenaelle Le Gall, David Vauzour

**Affiliations:** a Norwich Medical School, Faculty of Medicine and Health Sciences, University of East Anglia, Norwich, United Kingdom; b Institute of Dentistry, Faculty of Medicine & Dentistry, Queen Mary University of London, Blizard Institute, London, United Kingdom

**Keywords:** Microbiome, indoxyl sulfate, indole-3-propionic acid, 5-hydroxyindole acetic acid, choline, kynurenic acid, mild cognitive impairment, subjective cognitive impairment, gut-brain axis

## Abstract

**Introduction:**

A key component of disease prevention is the identification of at-risk individuals. Microbial dysbiosis in the early stages of cognitive decline and Alzheimer's disease (AD) and can modulate the levels of microbe-derived metabolites (MDM), thought to contribute to neuroinflammation, blood‒brain barrier dysfunction, and neuronal degeneration. However, the precise role of MDM in this process, as well as their potential value as risk factors, remains poorly understood.

**Methods:**

Mass spectrometry platforms determined the serum concentration of 33 metabolites (13 tryptophan-related compounds, 15 bile acid compounds, 3 TMAO-related metabolites and 2 cresol metabolites) from cognitively healthy subjects, subjective cognitive impairment (SCI) participants and mild cognitive impairment (MCI) participants (*n* = 50 per group, matched for age, BMI and sex). Multiple linear regression and machine learning techniques were applied to identify a metabolite panel capable of classifying early cognitive decline. 16S rRNA amplicon sequencing was employed to identify bacterial taxa associated with these metabolic changes.

**Results:**

Multiple linear regression modelling, adjusted for sex, BMI, age, albumin (for its role in metabolite transport), liver and kidney function, and background diet, identified key neuroprotective metabolites, namely choline, 5-hydroxyindole acetic acid, and indole propionic acid (IPA), as lower in SCI and MCI individuals compared to healthy controls. In contrast, the cytotoxic metabolite, indoxyl sulfate, and kynurenic acid were elevated. A random forest algorithm with multiclass classification further validated these findings, highlighting six metabolites (indoxyl sulfate, choline, 5-hydroxyindole acetic acid, IPA, kynurenic acid, and kynurenine) as classifiers of early cognitive decline, achieving an area under the curve (AUC) of 0.79.

**Conclusion:**

These findings suggest that MDM may serve as putative composite biomarkers of early cognitive decline, offering potential clinical relevance for metabolic risk stratification and supporting the future development of minimally invasive screening tools.

## 1. Introduction

Currently, an estimated 55.2 million people suffer from dementia worldwide, of which Alzheimer's disease (AD) is the main form.^1^ In the absence of an effective strategy to slow or prevent disease progression, dementia incidence is expected to increase to 152.8 million by 2050. By the time AD is typically diagnosed, substantial neuronal loss will have occurred across multiple brain regions. Identifying molecular precursors and biological risk factors associated with early cognitive decline, a clinical risk state for dementia including AD, would enable earlier detection and the targeting of regular monitoring and mitigating interventions while prevention is viable.

The contribution of lifestyle factors to cognitive decline and dementia is well documented.^2^^,^^3^ Diet in particular has emerged as a key influencer of brain health, in part by modulating communication along the microbiota‒gut–brain axis. This axis forms a bidirectional communication system comprising neuronal, endocrine, immune and metabolic signaling mechanisms linking the gut and the central nervous system (CNS).^4^ Gut microbes regulate this communication via the breakdown of dietary compounds into bioactive metabolites. Such microbe-derived metabolites (MDMs) can influence CNS function both directly, by crossing the blood‒brain barrier, and indirectly through effects on peripheral organ systems and modulation of vagal signaling.^5^ In the early stages of decline, for example, mild cognitive impairment (MCI), the microbiota–gut–brain axis becomes dysregulated (i.e., dysbiosis), a change associated with pathological processes such as neuroinflammation and neural injury, and thought to contribute to accelerating cognitive decline.^6-8^ However, the mechanism(s) underlying these changes, and the role of MDM in this process remains unknown.

Several examples of MDM have been linked to cognitive health,^5^ including trimethylamine *N*-oxide (TMAO),^9-12^ bile acids (BAs),^13-15^ tryptophan,^16-19^ *p*-cresol and its derivatives.^20^^,^^21^ Notably, these same MDMs have been further linked to pathological processes associated with AD,^21-28^ including neuroinflammation, synaptic damage and blood-brain barrier disruption, but whether changes in these MDMs are drivers or correlates of these processes requires comprehensive investigation. However, despite these links, many of these MDMs remain under-investigated in the context of early cognitive decline, where disease-modifying interventions may be most effective, representing a critical gap in the understanding of early metabolic dysregulation. As such, this study aimed to quantify and profile these metabolites to identify novel metabolic risk factors in early cognitive decline and inform early-detection and intervention strategies.

Targeted metabolomics presents a powerful tool to comprehensively assess changes in the endogenous metabolome. Here, we present a targeted metabolomics approach employing liquid chromatography-tandem mass spectrometry (LC-MS/MS) to quantify 33 metabolites, including 13 tryptophan-related compounds, 15 bile acid compounds, 3 TMAO-related metabolites and 2 cresol metabolites, in the serum of healthy controls and participants in early cognitive decline (e.g., individuals with subjective cognitive impairment (SCI) and mild cognitive impairment (MCI)). Multiple linear regression and machine learning techniques were applied to identify a metabolite panel to distinguish control, SCI and MCI participants. This study presents, for the first time, how key metabolites, in combination, can classify individuals at different stages of early cognitive decline. It is also one of the few studies characterizing metabolic perturbations in these early stages, including participants with SCI.

## 2. Materials and methods

### 2.1. Study samples

Overnight fasted human serum samples from the baseline measurements of two previously conducted clinical studies were used: (1) the impact of cranberries on the microbiome and brain in healthy aging studies (COMBAT; NCT03679533) and (2) the Cognitive Ageing, Nutrition and Neurogenesis (CANN; NCT02525198) study. The COMBAT study recruited 60 adults, aged 50-80 years, with no subjective memory complaints as assessed by the Cognitive Change Index (CCI) questionnaire.^29^ The CANN study recruited 259 participants, aged ≥ 50 years, with subjective cognitive impairment (SCI) or mild cognitive impairment (MCI) based on criteria developed by the National Institute of Aging-Alzheimer's Association, with no indication of clinical dementia.^30^ In brief, the classification of MCI was based on an established criteria, requiring (1) self-reported memory decline over 2–3 years, (2) preserved functional independence, indicated by a Functional Activities Questionnaire score of less than 6, (3) absence of dementia, defined as a Montreal Cognitive Assessment score of 18 or higher,^31^ and (4) no significant depression, determined by a Geriatric Depression Scale-15 score of less than 10.^32^ Additionally, MCI classification required cognitive impairment of at least one standard deviation below the age- and education-adjusted mean on at least one neuropsychological test assessing memory (California Verbal Learning Test-II,^33^ language (Boston Naming Test,^34^ visuospatial function (Figure Copy task of the Repeatable Battery for the Assessment of Neuropsychological Status,^35^ attention (Digit Span task, Forward or Backward, from the Wechsler Adult Intelligence Scale,^36^ or executive function (Trail Making Test, Part A or B.^37^ Participants meeting all criteria except for cognitive impairment were classified as SCI. Cognitively healthy adults were selected from the COMBAT study as a control group, with all groups (controls, SCI and MCI, *n* = 50 per group) matched for age, BMI and sex, as these are key variables known to affect microbiome composition.^38^^,^^39^

Participant recruitment for both the COMBAT and CANN studies followed consistent eligibility criteria which ensures baseline comparability. Individuals were eligible if they were aged ≥ 50 years, were generally healthy, fluent in English, and either cognitively intact or experiencing early cognitive concerns not meeting the criteria for dementia. Recruitment was restricted to residents of Norwich, UK, which provides geographical consistency, with data collection conducted by researchers from the same affiliated group using harmonized protocols. The overnight fasted blood sample was collected on participants' baseline visit for metabolomic and biochemical analyzes. The use of overnight fasted serum samples was implemented to minimize preanalytical variation, as metabolic markers fluctuate substantially following food intake.^40^ Fasting standardizes metabolic conditions across participants, thereby enhancing the reliability of metabolomic and biochemical analyzes and reducing the potential for dietary-induced variability to obscure metabolic patterns.

Fecal samples were collected during the baseline visit using the collection vessels (NHS-approved easy sampler collection kit supplied by Cover them Limited). The storage container was placed in a cool dry location prior to returning to the research facility at the earliest opportunity (i.e., at the study visit). Baseline biological samples from the CANN study were collected between 2015 and 2017 and stored for approximately 4–6 y, while baseline samples from the COMBAT study were collected between 2018 and 2019 and stored for approximately 2–3 y, prior to analysis. All samples were stored at −80 °C at the University of East Anglia Biorepository to preserve metabolite and microbiome integrity, a storage condition reported to maintain stability of the majority of plasma metabolites, with only approximately 2% exhibiting changes within the first seven years of storage.^41^ Following storage, all samples were processed in the same analytical batch for metabolomic and microbiome analyzes, minimizing technical variability and batch effects.

Biochemical analyzes included blood glucose, liver function (bilirubin, albumin, aspartate aminotransferase (AST), alanine aminotransferase (ALT) and AST/ALT ratio), kidney function (creatinine) and serum lipid concentrations (total-, LDL-, HDL-cholesterol and triglyceride) were conducted in all participants.

Exclusion criteria for both studies were designed to minimize confounding variables, particularly those affecting gut microbiota composition. Participants were excluded if they had a history of significant neurological, psychiatric, gastrointestinal, or metabolic disorders (e.g., diabetes mellitus, liver disease), chronic fatigue syndrome, or gall bladder abnormalities. Additional exclusions included current or recent smoking, alcohol or drug dependence, and use of medications or supplements known to alter gut microbiota (e.g., antibiotics, probiotics, prebiotics, symbiotics, fish oil, or high flavonoid intake, defined as >15 portions/d) within four weeks of sample collection. Individuals taking antidepressants, antipsychotics, anticoagulants (e.g., warfarin), or any medication affecting gastrointestinal function were also excluded. To further assess mental health, participants completed the patient health questionnaire-9 (PHQ-9) and the generalized anxiety disorder-7 (GAD-7).^42^ Those with clinically significant depression or anxiety were excluded in both studies. Participants reporting gastrointestinal symptoms such as diarrhea at baseline were also excluded.

Cognitive health was assessed using a variety of cognitive tests in both the COMBAT and CANN study at the participants' baseline visit. However, only the Trail Making Test (assessing visual processing speed, scanning, mental flexibility, as well as executive function) and the digit span test (assessing verbal short-term and working memory) were used across the COMBAT and CANN studies enabling comparisons. Participants also completed a validated, semi-quantitative Scottish Collaborative Group (SCG) food frequency questionnaire (version 6.6) to assess background diet.^43^

The protocols were approved by the UK National Research Ethics Service (NRES) Committee, (Study ID: 14/EE/0189) for CANN and by the University of East Anglia's Faculty of Medicine and Health Sciences Ethical Review Committee (Reference: 201819–039) and the UK Health Research Authority (IRAS number: 237251) for COMBAT. The participants provided written informed consent to participate.

### 2.2. Microbiome profiling

Microbiome analysis was performed by 16S rRNA amplicon sequencing as previously reported.^44^ In brief, DNA extraction was performed from approximately 50 mg of fecal content using the QIAamp PowerFecal Pro DNA Kit (Qiagen, Manchester, UK) as per the manufacturer's instructions. DNA quantity was evaluated using a Nanodrop 2000 Spectrophotometer (Fisher Scientific, UK). The quality assessment by agarose gel electrophoresis distinguished the DNA integrity, purity, fragment size and concentration. Illumina NovaSeq 6000 PE250 was used to amplify the V3–V4 hypervariable region. Sequence analysis was carried out using Uparse software (Uparse v7.0.1001),^45^ incorporating all the effective tags. Sequences sharing a similarity of ≥97% were grouped into the same operational taxonomic unit (OTU). A representative sequence for each OTU was screened for further annotation. A representative OTU sequence was further analyzed using the SSU rRNA database of SILVA Database 138.^46^ OTU abundance data were normalized using a standard sequence number corresponding to the sample with the least sequences. Alpha diversity was assessed using both Chao1 and Shannon H diversity indices, whilst beta diversity was assessed using Bray–Curtis dissimilarity. Statistical significance was determined by Kruskal–Wallis or permutational multivariate analysis of variance (PERMANOVA). Comparisons at the phylum and genus level were made using classical univariate analysis using Kruskal–Wallis combined with a false discovery rate (FDR) approach used to correct for multiple testing. *P*-values below 0.05 were considered statistically significant.

### 2.3. Metabolite profiling

Serum samples were diluted with methanol at a ratio of 1:10 (v/v) and placed on dry ice for 10  min. Samples were then centrifuged (5 min, 16,000× g at room temp), supernatants filtered using a 0.45 µM PTFE syringe filter and evaporated to dryness using a Savant™ SpeedVac™ high-capacity concentrator (Thermo Fisher, UK). Dried samples were resuspended in either 50 µL of methanol with the addition of 15 µL of lithocholic acid-d4 and cholic acid-d4 at 50 µg/mL for the detection of bile acids, 50 µL of water with TMA-d9 *N*-oxide, TMA 13 C 15 N hydrochloride at 50 µg/mL for the detection of TMAO/TMA/choline or 50 µL of water with 15 µL of L-methionine-3, 3, 4, 4 d4 and *p*-toluenesulfonic acid at 50 µg/mL for the detection of tryptophan and *p*-cresol metabolites respectively. All internal standards were supplied by Thermo Fisher, UK. Samples were analyzed using the Waters Acquity UPLC system and Xevo TQ-S Cronos mass spectrometer with MassLynx 4.1 software. See supplementary methods for full details.

### 2.4. Statistical analyzes

Orthogonal partial least squares–discriminant analysis (OPLS-DA) was performed using Metaboanalyst (v5.0), a web-based multivariate analysis tool.^47^ Significant associations of metabolites with cognitive status were identified using multiple linear regression analysis. Separate regression models were conducted for each metabolite as the outcome variable to assess potential associations with early cognitive decline. Covariates known to affect metabolome or microbiome composition, including age, BMI, diet and markers of kidney function (creatinine) and liver function (AST/ALT ratio), were included in the model.^48-52^ The model was also adjusted for albumin due to its role in the binding and transporting of key metabolites, including bile acids, cresols and indoxyl sulfate, potentially influencing their circulating levels.^53-55^ Diet was assessed using a validated, semiquantitative Scottish Collaborative Group (SCG) food frequency questionnaire (version 6.6).^43^ Participants' dietary components were grouped (kcal, proteins, fats, carbohydrates, water, alcohol, vitamins and minerals) and analyzed using hierarchical clustering via Ward's linkage method to assemble individuals with similar dietary patterns (Supplementary Figure S1). Ward's linkage method with Euclidean distance was used as it minimizes within-cluster variance, producing compact, interpretable clusters that capture cohesive dietary behaviors. This offered an advantage over methods such as PCA, which produce continuous components less suited for defining discrete dietary groups, and k-means, which is more sensitive to initial cluster centers. This clustered participants into low, moderate and high intake of dietary components and was added to the model as a categorical variable, with participants with a moderate intake used as a reference group. Age, BMI, creatinine, albumin and AST/ALT ratio were added to the model as continuous variables. Finally, sex and cognitive status (i.e., control, SCI and MCI) were added to the model as categorical variables. To minimize the influence of outliers, data points with values beyond ± 2 standard deviations from the mean were excluded, in line with previous blood metabolic studies.^56^ Outlier exclusion methods based on standard deviations minimize biases and maintain low Type I error rates compared to alternative methods.^57^ The assumptions for multiple linear regression analysis including the existence of a linear relationship among the outcome and predictor variable, normality and homoscedasticity were assessed (Supplementary Figure S2). The model tested for significant associations between metabolite and cognitive status, adjusting for the included covariates. All multiple linear regression analyzes were performed in R (v3.6.3; R Foundation: A Language and Environment for Statistical Computing).

### 2.5. Machine learning

A random forest (RF) machine learning algorithm was implemented to assess whether metabolites could be predictive of early cognitive decline. All machine learning models were built in Python (Python Software Foundation. Python Language Reference, version 3.8). Hyperparameters were optimized using the GridSearchCV package in scikit-learn via a 5-fold stratified cross-validation. The number of variables considered per split corresponds to the square root of the total number of attributes in the data^58^ (as 33 variables were considered, this resulted in ~6 random variables per split). To create a composite panel to predict early cognitive decline, metabolites were ranked according to the mean decrease Gini. This highlights the loss in model performance when permuting the predictor values, and can provide more robust results than mean decrease accuracy.^59^ The metabolites with the highest mean decrease Gini score producing the highest AUC values were retained in the model. To compare our model, Naive Bayes and AdaBoost machine learning models were also constructed.^60^^,^^61^ AdaBoost predictions were made by using a weighted average of weak classifiers. Our model contained 50 estimators, a learning rate of 1.00 and a SAMME classification algorithm which updated the base estimator's weights with classification results. The Naive Bayes method was applied based on applying Bayes' theorem with the “naive” assumption of conditional independence between every pair of features given the value of the target variable. The model used 5-fold cross-validation to provide a robust estimate of predictive performance. Models were evaluated using the macro-average area under the receiver operating characteristic curve (AUC), which measures overall performance across all classes by plotting the false positive rate against the true positive rate. Pairwise comparisons were also conducted to assess the model's ability to distinguish between groups. The same RF-based approach was subsequently applied to microbiome genera to investigate their potential to distinguish between groups, following the same feature selection and evaluation process.

## 3. Results

### 3.1. Study population characteristics

A total of 150 individuals were included in the study of which 50 (33.3%) were cognitively healthy, 50 (33.3%) presented with SCI and 50 (33.3%) with MCI. The mean ± SD age of all participants was 65.5 ± 5.7 y, with a mean level of education of 14.6 ± 3.5 y and 54% female (Table 1). Cognitive groups were matched for age, BMI and sex (*p* = 0.99). Participants in both the COMBAT and CANN study undertook several cognitive assessments at their baseline visit.^29^^,^^30^ Significant differences were found in the Trail Making Test B, digit span backward test and digit span total score between groups (*p* < 0.05). There was a marginal difference between the three groups in the trail-making test A (*p* = 0.09) and no significant difference occurred in the digit span forwards test (*p* = 0.21). The prevalence of the *APOɛ4* was lower in controls (18%) compared to SCI (26%) and MCI (38%) participants.

**Table 1.** Baseline characteristics of the participants.

	Control (*n *= 50)	SCI (*n *= 50)	MCI (*n *= 50)	*p*-value	Post hoc Tukey's Ttest
Sex, M/F (%F)	23/27 (54)	23/27 (54)	23/27 (54)	−	−
Age (years)	65.6 (5.3)	65.5 (6.1)	65.5 (5.8)	0.999	−
BMI (kg/m^2^)	25.1 (3.1)	25.0 (2.9)	25.0 (2.8)	0.993	−
Education (years)	14.4 (2.6)	14.6 (4.0)	14.6 (3.9)	0.968	−
% *APOE4*	18	26	38	0.079	−
**Cognitive tests**					
TMT A	30.7 (6.2)	29.3 (8.1)	33.3 (12.1)	0.088	−
TMT B	66.4 (20.4)	62.3 (16.5)	74.9 (27.3)	**0.015**	SCI < MCI (*p* = 0.012)
Digit span forwards	11.1 (2.2)	11.2 (1.8)	10.5 (2.6)	0.211	−
Digit span backwards	7.7 (2.0)	7.2 (1.8)	6.5 (2.1)	**0.011**	Control > MCI (*p* = 0.008)
Digit span total	18.8 (3.8)	18.4 (3.0)	17.0 (4.2)	**0.039**	Control > MCI (*p* = 0.043)
**Biochemistry**					
Creatinine (µmol/L)	73.90 (13.5)	72.5 (12.3)	73.5 (14.1)	0.871	−
Albumin (g/L)	40.4 (2.4)	30.0 (2.4)	39.4 (2.3)	**<0.001**	Control > SCI (*p* < 0.0001); SCI > MCI (*p* = 0.010)
Bilirubin (µmol/L)	12.8 (4.7)	8.9 (5.1)	9.1 (4.4)	**<0.001**	Control > SCI (*p* < 0.001); Control > MCI (*p* = 0.001)
AST (µL)	21.7 (3.9)	20.6 (5.9)	24.0 (13.1)	0.141	−
ALT (µL)	16.9 (5.4)	16.7 (9.0)	18.3 (11.3)	0.621	−
AST/ALT	1.4 (0.3)	1.4 (0.4)	1.4 (0.4)	0.548	−
Fasting Glucose (mmol/L)	4.8 (0.4)	5.0 (0.5)	5.3 (1.0)	**<0.001**	Control < MCI (*p* < 0.001)
Triglyceride (mmol/L)	1.1 (0.5)	1.1 (0.4)	1.2 (0.5)	0.368	−
Cholesterol (mmol/L)	5.6 (1.1)	5.2 (1.1)	5.2 (1.0)	0.174	−
HDL Cholesterol (mmol/L)	1.6 (0.4)	1.5 (0.5)	1.5 (0.4)	0.297	−
LDL Cholesterol (mmol/L)	3.4 (0.1)	3.2 (0.9)	3.1 (0.8)	0.182	−

Mean (SD). *p*-value calculated using one-way ANOVA. Significant values at *p* < 0.05 are in bold. SCI: subjective cognitive impairment, MCI: mild cognitive impairment, TMT: Trail Making Test A or B. Bold *p*-values represent *p* < 0.05.

Albumin, bilirubin and fasting glucose (*p* < 0.01) differed according to cognitive status. Interestingly, both albumin and bilirubin were highest in controls and lowest in SCI participants. Although participants diagnosed with diabetes mellitus were excluded, fasting glucose increased over control to SCI to MCI, with the lowest concentrations in control individuals and the highest in MCI (Table 1).

### 3.2. Gut microbiome and metabolome shifts in early cognitive decline

**Figure 1.**
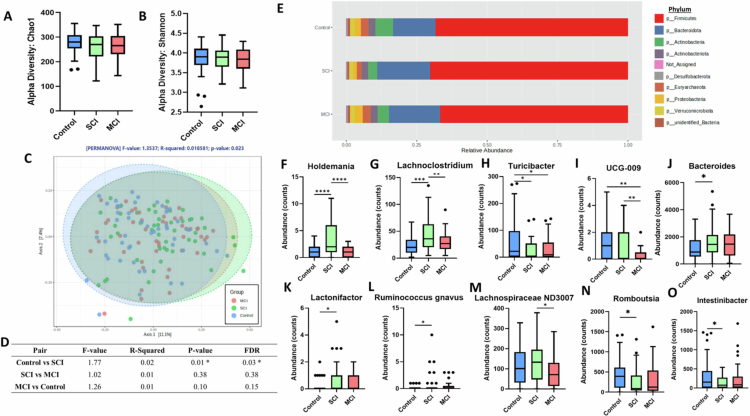
Fecal microbiome beta diversity is significantly altered in early cognitive decline. Alpha diversity measured by Chao1 (A) and Shannon H (B) index. (C) Beta diversity as measured by Bray‒Curtis; *p*-value generated from PERMANOVA. (D) Pairwise comparisons of the beta diversity analysis. (E) Relative abundance of the gut microbiome at phylum level. (F−O) Abundance counts of microbiome genera significantly (*p* < 0.05) modulated between control, SCI and MCI. * = *p* < 0.05, ** = *p* < 0.01.

Alpha diversity was measured using the Chao1 (*p* = 0.21) and Shannon H (*p* = 0.70) indices with no significant difference amongst groups (Figure 1A–B). Conversely, beta diversity, as measured by Bray‒Curtis dissimilarity, was significantly different (PERMANOVA F-value = 1.35, *p* = 0.02) (Figure 1C). Pairwise analysis suggested the shift was primarily driven by the differences between the control and SCI groups (FDR q = 0.03), rather than between SCI and MCI (FDR q = 0.38) or MCI and control (FDR q = 0.15) (Figure 1D). There was no significant difference in the gut microbiome at the phylum level (Figure 1E; Supplementary Table S1 for full abundance counts). However, 10 genera were modulated between groups (*p* < 0.05) (Figure 1F–O; Supplementary Table S2).

**Figure 2.**
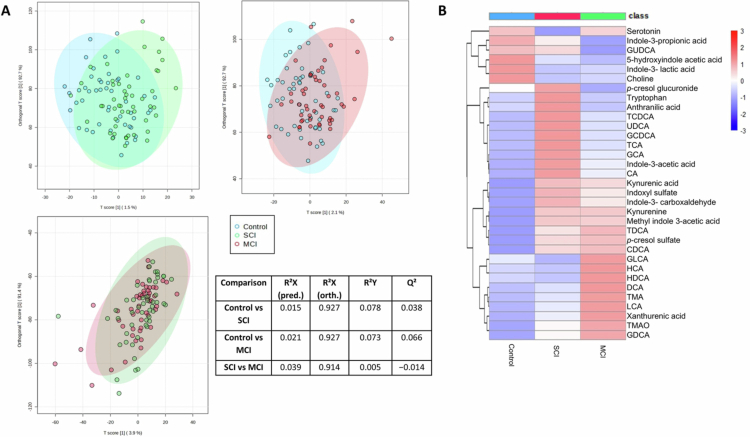
Metabolic shift occurs in early cognitive decline. (A) Orthogonal partial least squares discriminant analysis (OPLS-DA) plots of pairwise metabolomic profiles between control, SCI and MCI and model statistics (B) Heatmap displaying changes in concentrations of metabolites between the groups, with hierarchical clustering.

The metabolomic profiles of the control, SCI and MCI participants were analyzed using pairwise orthogonal partial least squares-discriminant analysis (OPLS-DA) (Figure 2A). OPLS-DA models comparing Control versus SCI and Control versus MCI demonstrated modest separation, whereas the OPLS-DA model comparing SCI and MCI displays minimal discrimination. The extent of separation between groups can be seen through the heatmap displaying shifts in metabolite concentrations between groups and clustering SCI and MCI together (Figure 2B). For full metabolite concentrations see Supplementary Table S3. Notably, changes observed in bile acid profiles were not attributable to differences in glycine or taurine conjugation (Supplementary Figure S3).

### 3.3. Serum dietary and microbial-derived metabolites assessed by targeted metabolomics reveal significant associations with gut microbiome alterations in early cognitive decline

**Figure 3.**
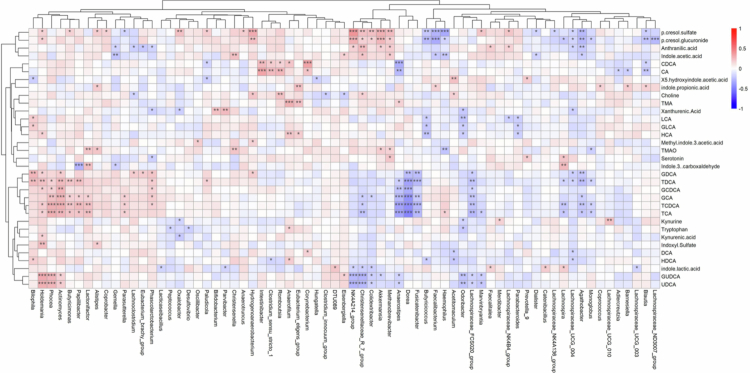
Serum metabolome and gut microbiome profiles are linked. Spearman rank correlation analysis between metabolite and microbiome genera. Red highlights a positive correlation and blue a negative correlation. * = *p* < 0.05, ** = *p* < 0.01, *** < 0.001.

Having identified a shift in both the microbiome and metabolome profiles, a Spearman rank correlation examined possible associations between the two datasets (Figure 3). This demonstrated a significant correlation between the 33 metabolites and 69 microbiota genera. For full correlation details, including R values, *p*-values, and FDR-adjusted *p*-values, see Supplementary Table S4. The similarity between microbiome and metabolomic profiles was confirmed by conducting a Procrustes analysis to evaluate the congruence of the two datasets. The analysis revealed significant similarity between the metabolome and microbiome results in control and SCI (R = 0.21, *p* = 0.03), SCI and MCI (R = 0.27, *p* = 0.002) and MCI and control (R = 0.26, *p* = 0.002) groups (Supplementary Figure S4).

### 3.4 Serum metabolites significantly associated with early cognitive decline in an adjusted multivariable model

Multiple linear regression analysis adjusted for liver function (AST/ALT ratio), kidney function (creatinine), age, BMI and background diet identified five metabolites significantly associated with early cognitive decline, including choline, 5-hydroxyindole acetic acid, indole-3- propionic acid (IPA), indoxyl sulfate and kynurenic acid (Table 2). Indoxyl sulfate, choline and 5-hydroxyindole acetic acid were associated with both SCI and MCI (*p* < 0.05). Kynurenic acid was significantly associated with SCI (*β* = 0.007, 95% CI: −0.006, 0.009, *p* = 0.015) but not MCI (*β* = 0.340, 95% CI: −0.006, 0.009, *p* = 0.735). On the other hand, IPA was significantly associated with MCI (*β* = −0.685, 95% CI: −1.121, −0.250, *p* = 0.002), but not SCI (*β* = −0.339, 95% CI: −0.810, 0.132, *p* = 0.157). Although IPA levels were lower in both SCI and MCI compared to controls, only MCI versus controls remained significant after adjusting for covariates, while kynurenic acid was higher in both groups but significantly associated only with SCI in the adjusted model, suggesting that these changes emerge at different stages of early cognitive decline. Neuroprotective metabolites, including choline, 5-hydroxyindole acetic acid and indole propionic acid,^62-64^ exhibited lower concentrations in SCI and MCI participants in comparison to controls, while metabolites linked to cytotoxicity, including indoxyl sulfate, showed increasing levels.^65^ Kynurenic acid was higher in SCI and MCI in comparison to controls.

**Table 2.** Multiple linear regression model (adjusted for age, BMI, sex, liver function (AST/ALT ratio), kidney function (creatinine), protein-bound transport (albumin), and diet) showing metabolites significantly associated with early cognitive decline.

	Metabolite																			
	Indoxyl sulfate				Choline				5-hydroxyindole acetic acid				Indole propionic acid				Kynurenic acid			
	*Beta*	*p-value*	*95% CI*		*Beta*	*p-value*	*95% CI*		*Beta*	*p-value*	*95% CI*		*Beta*	*p-value*	*95% CI*		*Beta*	*p-value*	*95% CI*	
Explanatory variable			*Low*	*High*			*Low*	*High*			*Low*	*High*			*Low*	*High*			*Low*	*High*
Constant	−3.913	0.471	−14.617	6.791	26.460	0.162	−10.714	63.635	0.147	0.006	0.044	0.250	4.047	0.085	−0.570	8.665	0.028	0.483	−0.050	0.105
Age	0.068	0.070	−0.006	0.142	0.115	0.378	−0.142	0.371	0.001	0.080	<0.001	0.001	−0.008	0.627	−0.040	0.024	0.000	0.546	−0.009	0.005
BMI	0.104	0.181	−0.049	0.257	−0.287	0.288	−0.817	0.244	−0.001	0.137	−0.003	<0.001	−0.042	0.210	−0.108	0.024	0.001	0.030	−0.013	0.005
Sex	−1.268	0.022	−2.354	−0.182	−1.298	0.497	−5.070	2.474	−0.013	0.016	−0.023	−0.002	0.364	0.126	−0.104	0.833	−0.006	0.135	−0.001	<0.001
Albumin	−0.032	0.724	−0.211	0.147	0.005	0.986	−0.617	0.627	−0.002	0.023	−0.004	0.000	−0.011	0.783	−0.088	0.066	−0.001	0.371	<0.001	0.002
Creatinine	0.065	0.001	0.027	0.103	0.129	0.054	−0.002	0.261	0.000	0.626	0.000	0.000	−0.006	0.437	−0.023	0.010	0.001	0.000	−0.014	0.002
AST/ALT	−1.717	0.003	−2.860	−0.574	0.776	0.699	−3.192	4.745	−0.006	0.322	−0.017	0.005	0.226	0.366	−0.267	0.719	−0.009	0.029	−0.002	0.001
Diet Group 1	0.257	0.599	−0.707	1.221	0.945	0.578	−2.404	4.294	−0.005	0.303	−0.014	0.004	0.224	0.289	−0.192	0.640	−0.002	0.517	<0.001	0.001
Diet Group 3	0.001	0.999	−1.233	1.235	−0.901	0.678	−5.186	3.385	0.002	0.692	−0.009	0.014	0.217	0.423	−0.316	0.749	−0.004	0.416	−0.017	−0.001
SCI	1.824	**0.001**	0.732	2.915	−5.514	**0.005**	−9.306	−1.723	−0.019	**<0.001**	−0.030	−0.009	−0.339	0.157	−0.810	0.132	0.007	**0.047**	−0.001	0.015
MCI	1.336	**0.010**	0.327	2.345	−5.142	**0.004**	−8.647	−1.638	−0.021	**<0.001**	−0.030	−0.011	−0.685	**0.002**	−1.121	−0.250	0.340	0.735	−0.006	0.009
Change in early cognitive decline	Increased				Decreased				Decreased				Decreased				Increased			

Diet was analyzed using hierarchical clustering, ‘Ward’ method, to group individuals with similar dietary patterns. This grouped participants into three dietary groups (low, moderate and high intake of dietary components (Kcal, carbohydrates, fats, protein, water, alcohol, minerals, vitamins). Healthy controls and diet group 2 (moderate intake) were used as reference groups in the model. Bold p-values represent *p* < 0.05 within SCI and MCI.

### 3.5 Machine learning models to identify metabolic risk factors distinguishing between stages of early cognitive decline

**Figure 4.**
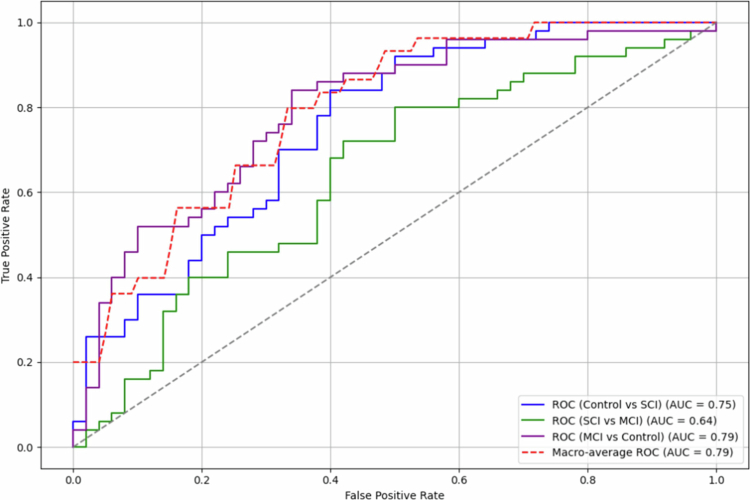
Six circulatory metabolites can distinguish stages of early cognitive decline. Receiving operating characteristic (ROC) curve illustrating the performance of the random forest model for both pairwise (solid lines) and multilevel (dashed line) classifier of controls, SCI and MCI participants. AUC = area under the curve. The six metabolites included in the model were: 5-hydroxyindole acetic acid, indole-3-propionic acid, choline, indoxyl sulfate, kynurenic acid, and kynurenine.

All 33 serum metabolites were initially evaluated as possible predictors of early cognitive decline. RF achieved the highest macro average multiclass classification AUC of 0.65, with AdaBoost and Naïve Bayes attaining 0.58 and 0.63, respectively (Supplementary Table S5). Using the mean decrease Gini, the importance of each metabolite was assessed (Supplementary Figure S5). Six metabolites (5-hydroxyindole acetic acid, indole-3-propionic acid, choline, indoxyl sulfate, kynurenic acid and kynurenine) produced the highest multiclass classification AUC of 0.79 using the RF classification algorithm (Figure 4). In comparison, Naive Bayes achieved an AUC of 0.72 and AdaBoost attained 0.68. Pairwise comparisons showed that the RF model achieved an AUC of 0.75 for control vs. SCI, 0.64 for SCI vs. MCI, and 0.79 for MCI vs. control, suggesting classification performance was strongest for distinguishing control from MCI. Additional metrics, i.e., recall, precision, and F1 score can be found in Supplementary Table S6. Due to these findings, we investigated whether model performance would be improved by predicting only healthy ageing and MCI. Using the six serum metabolites from controls and MCI participants, the RF model showed improved predictive performance (AUC = 0.84). AdaBoost and Naive Bayes also demonstrated increased performance, attaining AUCs of 0.87 and 0.90, respectively (Supplementary Table S5).

A separate RF model was constructed to evaluate the ability of microbiome genera to distinguish stages of early cognitive decline. Similar to the metabolite analysis, all genera were initially assessed. The importance of each genus was evaluated using the mean decrease Gini, identifying the top eleven genera, *Clostridium sensu stricto, Romboutsia, Christensenellaceae R 7 group, Ruminococcus torques group, Oscillibacter, Eubacterium eligens group, Lachnoclostridium, Anaerostipes, Ruminococcus, Holdemania, Butyricicoccus* and *Intestinibacteras,* being the most predictive (Supplementary Figure S6). Selected genera produced the highest multiclass classification AUC of 0.59 using the RF classification algorithm. Pairwise comparisons demonstrated that the RF model achieved AUCs of 0.66 for control vs. SCI, 0.65 for SCI vs. MCI, and 0.61 for MCI vs. control, respectively. However, it did not achieve as high an AUC as the metabolome panel in classifying the three groups, likely due to circulatory metabolites being influenced by host biosynthetic mechanisms, which regulate metabolite levels and serve as key intermediaries in the gut–brain axis.^5^ Additional metrics, i.e., recall, precision, and F1 score can be found in Supplementary Table S6.

## 4. Discussion

Identification of robust, inexpensive, and noninvasive markers of cognitive status and its trajectory is currently an unmet need in dementia research, particularly at stages of early cognitive decline associated with increased risk of AD. Emerging evidence suggests that circulatory metabolites produced or modulated by gut microbiota can impact brain function through the microbiota–gut–brain axis. Metabolic alterations contain rich systemic information on the underlying physiology that connects the periphery to the CNS, likely affecting numerous pathways simultaneously. Thus, the simultaneous detection of perturbed metabolites can provide a powerful detection tool. However, studies investigating composite markers are lacking.

16 s rRNA amplicon sequencing indicated that significant shifts in gut microbiome composition occur during early cognitive decline, commencing as early as SCI, suggesting changes may already be apparent when memory complaints first appear, aligning with previous studies.^66^^,^^67^ As cognitive decline progresses from SCI to MCI, gut microbiome modulation appears less significant. This could indicate greater heterogeneity within the MCI group. Recent evidence has shown that individuals with MCI follow distinct cognitive trajectories, ranging from stable to rapidly declining, each associated with differing AD biomarker profiles.^68^ Heterogeneity may dilute group-level signals when comparing MCI participants to controls, making it harder to detect consistent microbiome shifts. Moreover, it aligns with previous studies reporting no significant differences in microbiome composition between MCI and control groups.^69^^,^^70^ However, these studies did not examine SCI, a less explored stage in microbiome research. Individuals with SCI are likely at a higher risk of cognitive decline progression compared to those who are cognitively healthy,^71^ which may lead to greater alterations in biological markers, including the gut microbiome and its metabolites.

Several genera identified as differentially abundant in early cognitive decline have previously been implicated in neurocognitive health. Elevated *Bacteroides* has been associated with increased neuroinflammation and amyloid pathology in AD cohorts, often correlated with markers such as CSF YKL‑40.^72^ Higher *Ruminococcus gnavus* levels have been implicated in inflammatory responses and were observed to correlate with cognitive decline, including in AD mouse models.^73^ Lower *Turicibacter* abundance has been observed in AD, and its reduction correlates with elevated biomarkers of glial activation, supporting a possible role in microbiota‒gut‒brain axis dysfunction.^72^ *Holdemania*, part of the *Erysipelotrichaceae* family, has been linked to age-related inflammation and metabolic dysregulation in older adults,^74^ though specific cognitive associations remain to be clarified. Procrustes analysis showed significant congruence of the microbiome and metabolome datasets, suggesting the two are interlinked and provides the potential for MDM risk factors to be detected early in disease progression. Indeed, gut microbiome composition can account for up to 58% of the variation of circulatory metabolites communicating along the microbiota‒gut‒brain axis.^75^

Targeted metabolomics quantifies metabolites with extremely high sensitivity and accuracy, providing an advantage over the relative responses yielded by untargeted approaches. RF and multiple linear regression models both revealed indoxyl sulfate, choline, 5-hydroxyindole acetic acid, IPA and kynurenic acid as key early indicators of cognitive decline, with RF presenting an AUC predictive performance of 0.79, strongly supporting a significant link between metabolic perturbations associated with the gut microbiome and early cognitive decline. Previous studies have predominantly concentrated on binary classification approaches, primarily utilizing MRI and PET imaging modalities, to investigate AD progression.^76-78^ However, in clinical practice, multiclass classification of blood samples of patients with SCI, MCI and healthy controls could provide a useful approach. Tong and colleagues attained a similar predictive performance (AUC = 0.73) using RF and nonlinear graph fusion of multiple modalities (regional MRI volumes, voxel-based FDG-PET signal intensities, CSF biomarker measures and genetic information) to classify control, MCI and AD participants.^79^ AUC increased to 0.87 when predicting healthy ageing and MCI, likely due to the difficulty of diagnosing a patient undergoing SCI. Indeed, Purser and colleagues found no relationship between memory complaints and the progression of cognitive impairment over 10 years in individuals 65 years and over.^80^ However, others dispute this result.^81^ Adjusting our statistical analysis for confounding variables that heavily influence the host, such as age, BMI, sex, albumin, kidney function, liver function and background diet, improves analysis robustness and sensitivity. Adjusting for background diet becomes particularly vital when examining MDM as the diet can both modulate gut microbiome composition and provide a variety of bioactive precursor compounds; a factor which is often overlooked in metabolomic analyzes.^82^ Nevertheless, our results highlight the use of profiling select circulatory MDM to identify higher-risk individuals of cognitive decline.

Of the five metabolites highlighted by both machine learning and multiple linear regression, all except choline are produced from tryptophan metabolism, indicating notable alterations in tryptophan metabolism may occur in early AD progression. Tryptophan metabolism has previously been well-linked to cognitive decline and AD.^18^ Indeed, we find lower neuroprotective tryptophan-derived metabolites, including IPA and 5-hydroxyindole acetic acid, as cognitive decline progresses from controls to SCI and MCI. IPA is produced in the gut by the microbial conversion of tryptophan via the indole pathway and has previously been investigated as a possible treatment for AD^83^ due to its potent antioxidant effect against Aβ 1-42 *in vitro*^84^ and its ability to prevent aggregation and deposition of Aβ monomers.^85^ IPA is anti-inflammatory, reducing the concentration of the proinflammatory TNF-*α* in activated microglia,^86^ lowering the expression of chemokine (CC Motif) ligand 2 (CCL2) and nitric oxide synthase 2 (NOS2) in interferon-beta (IFN-*β*) activated murine astrocytes^87^ and preventing increases in cytokines in LPS-induced human primary astrocytes,^88^ and has previously been identified as a predictor of AD progression.^89^ 5-hydroxyindole acetic acid is often used as a surrogate marker for serotonin due to serotonin's rapid degradation. As such, our findings indicate lower peripheral serotonin breakdown as early cognitive decline progresses. Approximately 95% of all serotonin is localized in peripheral compartments where it is involved in the modulation of the enteric nervous system (ENS) development and neurogenesis, gut motility, secretion, inflammation, and epithelial development, suggesting these processes may be disrupted in early cognitive decline.^90^ Indeed, MCI and AD patients have often been reported to suffer from gastrointestinal symptoms^91^ and ENS dysregulation in AD has previously been described.^92^ Decreased concentrations of 5-hydroxyindole acetic acid also suggest a shift in tryptophan metabolism towards the kynurenine pathway, reducing the availability of tryptophan for serotonin synthesis. This is supported by higher serum kynurenine concentrations in SCI and MCI participants in comparison to controls (Supplementary Table S3) and has previously been found in AD participants, linked to poor memory, executive function and global cognition.^93^ The kynurenine pathway is activated by an inflammatory stimulus, promoting indoleamine 2,3-dioxygenase, the rate-limiting enzyme that initiates the kynurenine pathway. Increased inflammation is a common feature of AD and as such may play a role in modulating tryptophan catabolites.

Both indoxyl sulfate and kynurenic acid concentrations were increased as cognitive decline progressed, even after adjusting for measures of liver and kidney function. As a uremic toxin, indoxyl sulfate can disrupt neuronal efflux transport systems, promote the production of free radicals, inflammation, endothelial cell dysfunction and disturb the circadian rhythm involved in clearing renal and CNS toxins,^94^^,^^95^ likely contributing to cognitive decline. Serum levels of indoxyl sulfate, as well as albumin, have previously been identified as predictors of cognitive impairment in participants with end-stage renal disease.^96^ End-stage renal disease patients exhibit significantly elevated indoxyl sulfate levels due to impaired renal clearance, alongside an increased abundance of the gut bacteria *Holdemania*, in line with our findings.^97^ Increased abundance in end-stage renal patients suggests it may contribute to indoxyl sulfate levels through direct or indirect mechanisms, possibly through involvement in tryptophan metabolism.^98^^,^^99^ Rodent studies show increased kynurenic acid concentrations can impair cognitive function, including spatial working memory, and broad monitoring deficits.^100^^,^^101^ However, data regarding this relationship in human studies is inconsistent.^102^^,^^103^ Kynurenic acid can play a protective role against the cytotoxic product of the kynurenine pathway, quinolinic acid, by acting as an NMDA antagonist for both glycine and glutamate modulatory sites.^104^ However, abnormal accumulation has previously been found to induce glutamatergic hypofunction and subsequently disrupt cognitive function.^105^ In AD, increased blood concentrations of kynurenic acid have been hypothesized to relate to neuroinflammatory processes and may be produced as a protective response to neuronal damage.^106^

Choline is required for numerous biological functions in the body,^107^ notably including hallmark AD-associated processes such as acetylcholine synthesis.^108^ As choline readily crosses the blood-brain barrier, peripheral concentrations typically mirror concentrations in the brain,^109^ thus lower concentrations in early cognitive decline may indicate decreased central acetylcholine production. Acetylcholine is intricately connected to neural networks regulating memory, and a reduction in this system is closely associated with learning and memory deficits in AD.^110^ *Lachnoclostridium* and *Lactonifactor* were inversely correlated with choline levels, suggesting changes in these genera may modulate blood concentrations. Indeed, previous research has found *L. saccharolyticum* WM1, a representative strain of *Lachnoclostridium*, to be an efficient converter of choline to TMA *in vitro*, transforming at a rate near 100%.^111^ This metabolic process *in vivo* also elevated serum TMAO levels,^111^ which is supported by our results displaying a 1.6-fold higher TMAO in MCI compared to controls. It is likely that increases in *Lachnoclostridium* abundance may increase the metabolism of choline to TMAO, decreasing its concentration in circulation.

Our study has major strengths including simultaneously targeting some of the top microbial and metabolic metabolites associated with cognitive decline, while matching our participants and adjusting our analysis for key factors known to influence the metabolome (age, BMI, sex, liver function, kidney function and background diet), factors rarely accounted for in marker studies. Furthermore, our study highlights key microbiota associated with these metabolic changes, as well as investigating participants from the earliest stage of decline (SCI) and validating our results through machine learning and adjusted statistical approaches. However, some limitations should be stressed. Despite our study adjusting results for key covariates, host metabolome profiles are influenced by a plethora of additional largely environmental and biological factors. Thus, although our findings suggest relationships between the variables, we cannot infer causal relationships from this analysis alone. Additionally, while Spearman correlation identifies potential associations between metabolites and the microbiome, it cannot establish causality and determine whether microbial changes directly influence metabolite levels or if the relationships are driven by other confounding factors. Furthermore, running separate regressions for each metabolite without correction for multiple comparisons may increase the risk of false positives. However, given the exploratory nature of this study and the sample size, sensitivity to detect potential biologically relevant signals was prioritized. Moreover, participants' background diet was adjusted for using data collected by food frequency questionnaires, which can be prone to measurement error and may introduce inaccuracies due to recall bias and self-reporting issues. While we excluded participants with diagnosed depression or anxiety to minimize confounding effects related to mood disorders, conditions known to impact tryptophan metabolism and inflammation, we recognize that some of the observed metabolic shifts may not be entirely specific to SCI/MCI. Given that tryptophan metabolism plays a significant role in broader inflammatory responses, it is possible that the observed alterations could reflect a physiological state associated with systemic inflammation rather than being exclusive to early cognitive decline. However, as neuroinflammation plays a critical role in cognitive decline, these findings remain relevant for understanding disease mechanisms and identifying potential risk factors of early disease progression. Furthermore, like all studies utilizing machine learning models, the larger the dataset, the more robust the predictive performance. With the current dataset including 150 individuals and 33 metabolites, we achieved significant predictive performance. However, these findings are hypothesis-generating and will require external validation in larger independent cohorts to improve the model.

## 5. Conclusion

Pathophysiological changes associated with neurodegenerative processes can begin many years prior to the onset of overt clinical symptoms, highlighting the importance of prevention research aimed at identifying novel risk factors underlying early cognitive decline. Scalable markers that enable the early detection of at-risk persons could permit the targeting of lifestyle interventions to lessen future risk and uncover novel mechanisms underpinning dementia. We signify a major role for the gut in connection to the brain through the modulation of key MDM. Furthermore, we lend strength to the hypothesis that individuals with higher risks of cognitive decline can be identified via a targeted metabolomic approach in the early stages of cognitive decline. Future research should focus on validating these metabolite signatures in larger, independent cohorts and exploring their potential for guiding early interventions to slow or prevent cognitive decline.

## Supplementary Material

Supplementary_Connell et al_Risk Factors ms_rev3.docx Supplementary_Connell et al_Risk Factors ms_rev3.docx

Supplementary material Supplementary Table S4

## Data Availability

The 16S rRNA gene sequence data have been deposited in the NCBI BioProject database (https://www.ncbi.nlm.nih.gov/bioproject/) under accession number PRJNA1109848. Other data that support the findings of this study are available from the corresponding authors upon reasonable request.
