# Supplementary material for: Circulatory dietary and gut-derived metabolites predict early cognitive decline
Source: Gut Microbes. 2026 Mar 27;18(1):2649487. doi: 10.1080/19490976.2026.2649487 (PMC13034628; doi:10.1080/19490976.2026.2649487)
Supplement: Supplementary_Connell et al_Risk Factors ms_rev3.docx [file KGMI_A_2649487_SM3838.docx]

**Supplementary data**

**Circulatory dietary and gut-derived metabolites predict prodromal Alzheimer's disease.**

Emily Connell^1^, Saber Sami^1^, Mizanur Khondoker^1^, Anne-Marie Minihane^1^, Matthew G. Pontifex^1^, Michael Müller^1^, Simon McArthur^2^, Gwenaelle Le Gall^1†^ and David Vauzour^1†^*

^1^ Norwich Medical School, Faculty of Medicine and Health Sciences, University of East Anglia, Norwich NR4 7TJ, United Kingdom.

^2^ Institute of Dentistry, Faculty of Medicine & Dentistry, Queen Mary University of London, Blizard Institute, London E1 2AT, United Kingdom

† These authors share senior authorship

***** To whom correspondence should be addressed: Dr David Vauzour; email: [D.Vauzour@uea.ac.uk](mailto:D.Vauzour@uea.ac.uk)

**1. Supplementary Methods of LC-MS/MS**

1.1 Sample collection

Overnight fasted blood samples were drawn from participants during their baseline study visit of the COMBAT and CANN studies. After collection, blood was left to coagulate (in clot-activating gel tubes), followed by centrifugation at 2,000g for 10 min and removal of the resultant serum from the sub-fractions. Aliquoted serum was stored at −80°C until further analysis.

1.2 Sample preparation

Serum samples were diluted with ice-cold methanol at a ratio of 1:10 (*v/v*) and placed on dry ice for 10 min. Samples were centrifuged (5 min, 16,000x g at room temp), supernatants filtered using a 0.45 µM PTFE syringe filter and evaporated to dryness using a Savant™ SpeedVac™ High-Capacity Concentrator (Cat. SC210A-230). For the detection of bile acids, dried samples were resuspended in 50 µL of methanol with 15 µL of lithocholic acid-d4, cholic acid-d4 at 50 µg/mL as the internal standards. For the detection of TMAO/TMA/choline, dried samples were resuspended in 50 µL water with TMA-d9 N-oxide, ^13^C_3_^15^N TMA hydrochloride at 50 µg/mL as the internal standards. Finally, for the detection of tryptophan and *p-cresol-related* metabolites, dried samples were resuspended in 50 µL water with 15 µL of L-methionine-3, 3, 4, 4 d4 and p-toluenesulfonic acid at 50 µg/mL as the internal standards for tryptophan and *p*-cresol metabolites respectively.

Stock solutions of each metabolite were prepared in methanol (1mg/mL) and stored at -80°C. Calibration standards were prepared by pooling all relevant analytes for each method at eight concentrations and adding the respective internal standards at 50 µg/mL. Calibration standards were run at the beginning, middle and end of each analytical queue. The analyte: internal standard response ratio was used to create calibration curves and quantify each metabolite.

1.3 LC-MS/MS Condition

Metabolite quantification was performed using liquid chromatography-tandem mass spectrometry (LC-MS/MS) comprising of Waters Acquity UPLC system and Xevo TQ-S Cronos mass spectrometer controlled by MassLynx 4.1 software. For the detection of bile acids, the electrospray ionisation (ESI) operated in negative mode and chromatographic separations were performed with a Supelco Ascentis Express C18 column (150 x 4.6 mm, 2.7 µM) (adapted from ^1^). Eluent A (10mM ammonium acetate, 0.1% formic acid, water) and eluent B (10mM ammonium acetate, 0.1% formic acid, methanol ran at a constant rate of 0.6 mL/min. The gradient began at 50% B and was held for 2 min before a linear increase to 95% B occurred at 20 min. This was held for 4 min before a linear decrease in gradient back to 50% B occurred between 24 and 25 min. The gradient was held at 50%B for another 4 mins.

For the detection of TMAO, TMA and choline, ESI operated in positive mode (adapted from ^2,3^). Chromatic separation occurred using a BEH Amide (150 x 2.1 mm, 1.7 µM) and 0.5mL/min using eluent A (10mM ammonium formate, 0.2% formic acid, 50% acetonitrile) and eluent B (10mM ammonium formate, 0.2% formic acid, 95% acetonitrile) initially at 100% B to 60% B at 4 min and held until 4.2 min, before increasing back to initial conditions of 100% B at 4.21 min and being held until 5.4 min for equilibration.

ESI operated in positive mode for the detection of tryptophan and *p-cresol-related* metabolites and chromatic separation occurred using an ACQUITY UPLC BEH C18 1.7 µM (2.1 x 50mm) column at a rate of 0.3 mL/min and composition of eluent A (0.1% formic acid, water) and eluent B (0.1% formic acid, methanol) at a gradient of 5% B from 0 to 0.5 min, 10% B from 0.5 to 2.5 min, 15% B at 3.5 min, 35% B at 4.5 min, 45% B at 6.5 min, 55% B at 7 min, 100% B at 7.5 min, 100% B until 10 min, and 5% B at10.1 min to return to initial conditions for equilibration until 14 mins. This method was adapted from Anesi and colleagues ^2^. Chromatogram peak analysis was performed by the accompanying Waters® TargetLynx ™ application manager and all further data analysis and calibration curve constructions were completed in Microsoft Excel (2019 version).

1.4 Method Performance

Excellent linear response range was ensured for each calibration curve with correlation coefficients (r^2^) 0.99 or higher for all calibration curves generated. An agilent high-performance autosampler with an injection program was used to minimise carry-over effects between samples. Samples were run in a random order and 20% of the whole set was re-run as a quality control for the method repeatability. No signal was also detected in blank samples run amongst the serum and calibration injections, or in blanks run after the highest calibration standard, indicating that there was little to no carry-over occurring.

**Supplementary Figures**


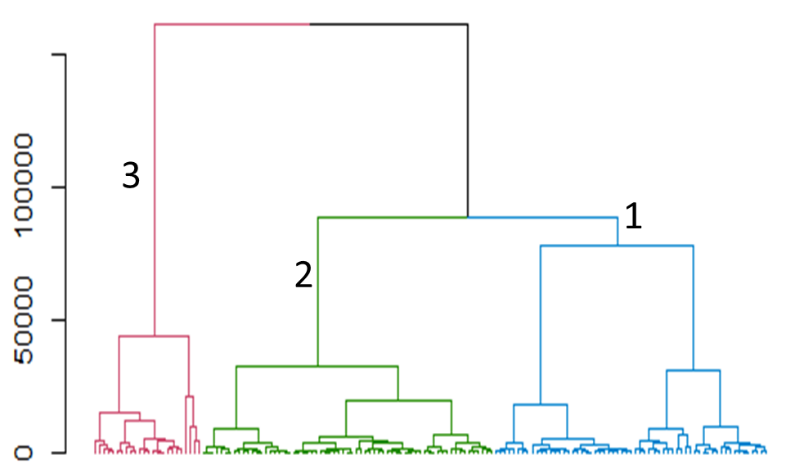


**Supplementary Figure S1: Hierarchical clustering of participant food frequency questionnaires.** Food frequency questionnaires were analysed using hierarchical clustering ‘Ward’ method to cluster participants with similar dietary patterns. This grouped participants into low intake of macronutrients (1), moderate intake of macronutrients (2) and high intake of macronutrients (3).


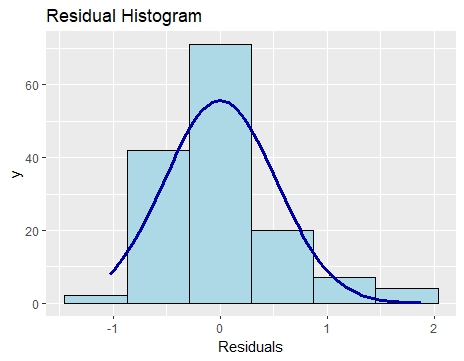

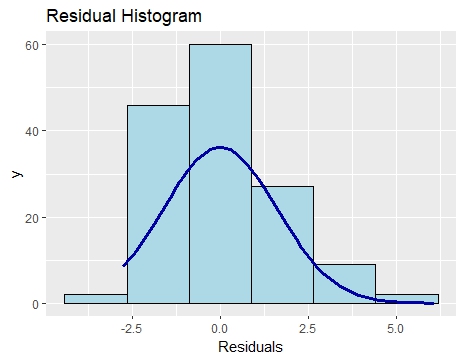

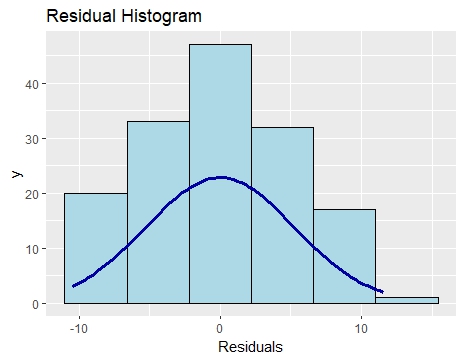

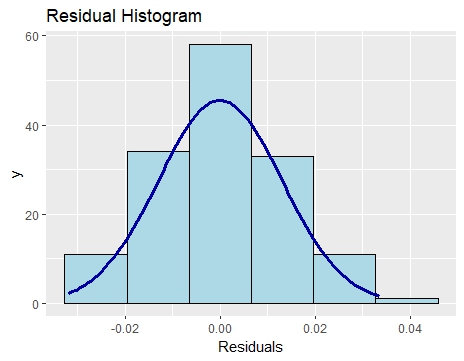

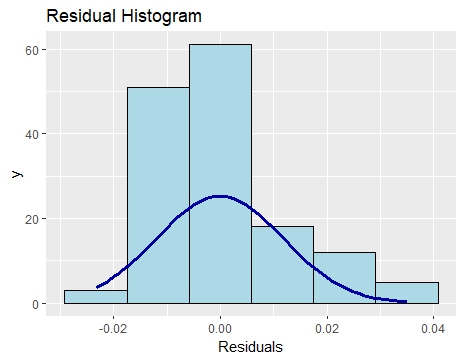


C

B

A

E

D


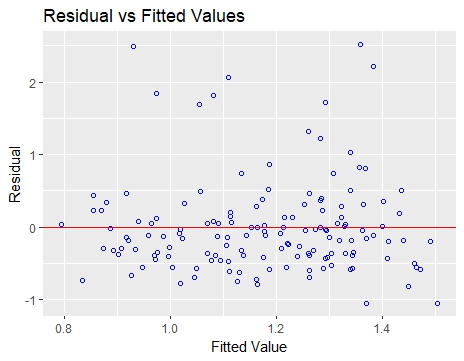
**
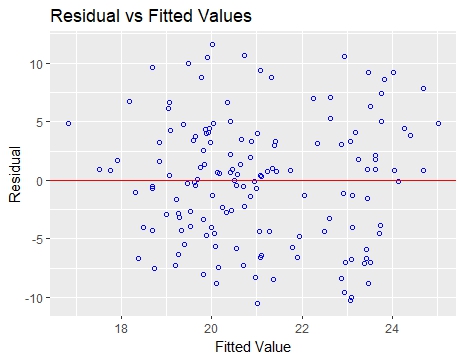
**
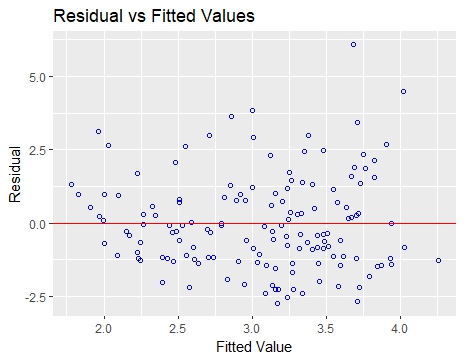

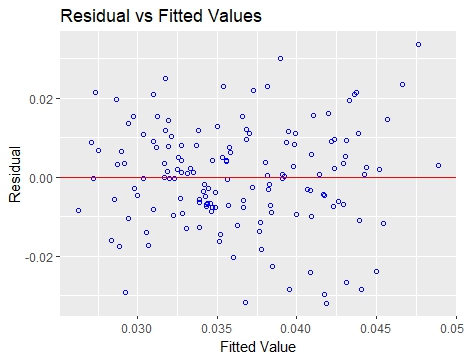


H

G

F


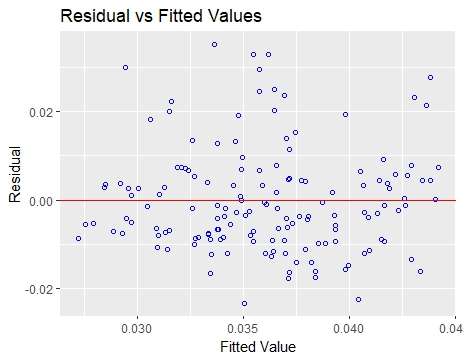


J

I

**Supplementary Figure S2: Assumptions of the multiple linear regression analysis**. Histogram displaying normality of the residuals for (A) choline, (B) indoxyl sulfate, (C) indole propionic acid, (D) kynurenic acid, (E) 5-hydroxyindole acetic acid. Residuals versus fitted plot confirming homoscedasticity of (F) choline, (G) indoxyl sulfate, (H) indole propionic acid, (I) kynurenic acid, (J) 5-hydroxyindole acetic acid.


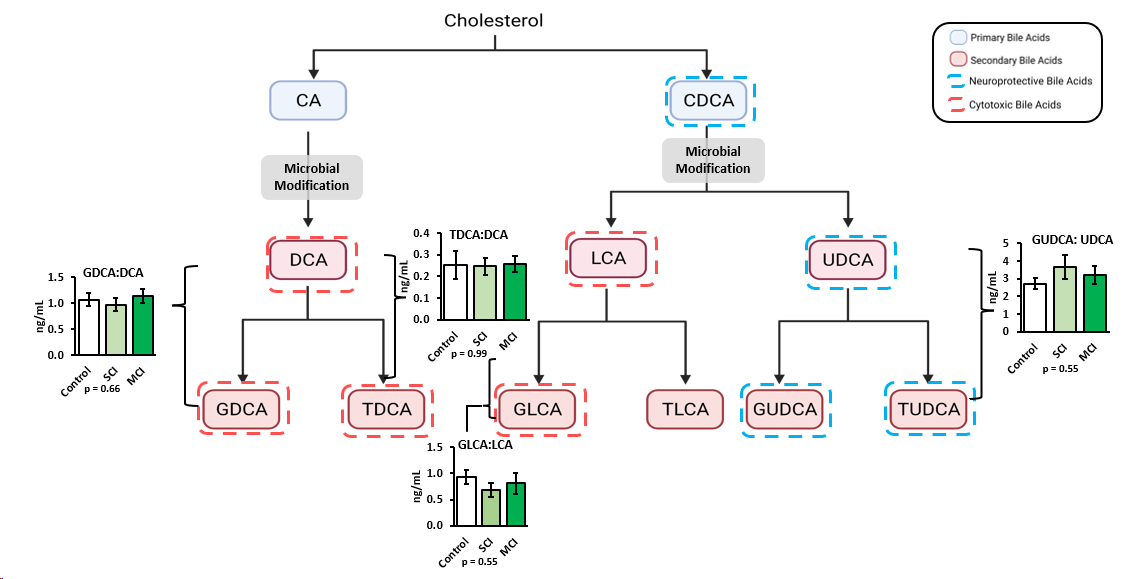


**Supplementary Figure S3:** Changes in bile acids were not due to modulation of glycine and taurine conjugation.


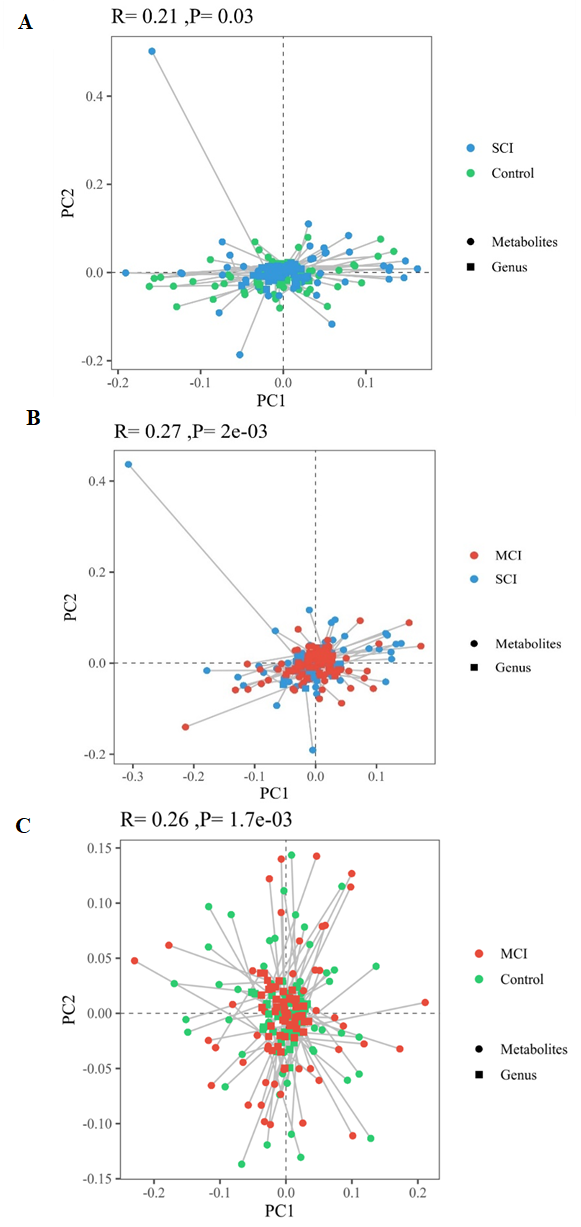


**Supplementary Figure S4: Serum metabolites and microbiome composition are significantly linked**. Procrustes plot comparing the relationship between the microbiome and the metabolome profiles in control and SCI (A), SCI and MCI (B) and MCI and control (C). Longer lines indicate more within-subject dissimilarity.


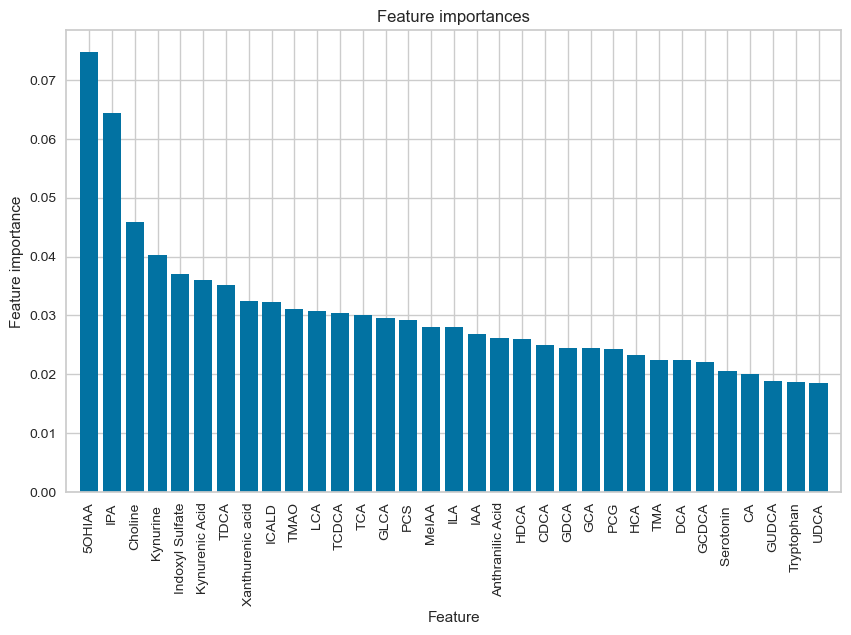


**Supplementary Figure S5: Identification of the top metabolites predictive of early cognitive decline**. Mean decrease Gini values highlighting the importance of each metabolite in our model. Red box indicates the top six metabolites that gave the highest AUC scores


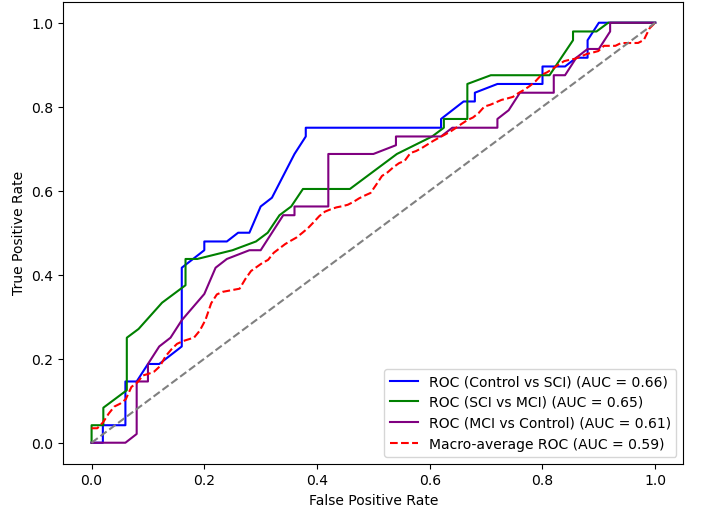
**Supplementary Figure S6: Receiving operating characteristic (ROC) curve of top eleven microbiome genera distinguishing stages of prodromal AD.**  Random Forest model for both pairwise (solid lines) and multilevel (dashed line) classifier of controls, SCI and MCI participants. AUC= area under the curve.

**Supplementary Tables**

**Supplementary Table S1: Gut microbiome abundance in control, MCI and SCI at the phylum level.** P-value generated by one-way ANOVA, with false discovery rate (FDR) correction for multiple testing.

| **Phylum** | **Control**  **(n=50)** | | **SCI**  **(n=50)** | | **MCI**  **(n=50)** | | **F Value** | **P-value** | **FDR** |
| --- | --- | --- | --- | --- | --- | --- | --- | --- | --- |
|  | **Mean** | **SD** | **Mean** | **SD** | **Mean** | **SD** |  |  |  |
| Unidentified Bacteria | 922.44 | 594.05 | 650.33 | 501.09 | 935.52 | 937.25 | 2.543 | 0.082 | 0.572 |
| Actinobacteria | 6256.20 | 9912.81 | 3364.15 | 3993.76 | 4297.44 | 5447.30 | 2.202 | 0.114 | 0.572 |
| Bacteroidota | 15959.58 | 9704.01 | 18935.69 | 10742.37 | 18767.63 | 10264.87 | 1.315 | 0.272 | 0.637 |
| Desulfobacterota | 225.88 | 282.13 | 150.90 | 201.20 | 233.40 | 330.38 | 1.313 | 0.272 | 0.637 |
| Euryarchaeota | 2614.94 | 5094.50 | 1686.13 | 3064.45 | 2911.29 | 4357.00 | 1.079 | 0.343 | 0.637 |
| Proteobacteria | 2370.36 | 6826.07 | 1382.56 | 2843.53 | 3258.67 | 8728.70 | 0.968 | 0.382 | 0.637 |
| Not Assigned | 341.88 | 217.19 | 321.08 | 122.14 | 325.19 | 134.15 | 0.222 | 0.802 | 0.989 |
| Actinobacteriota | 2437.84 | 2206.50 | 2297.54 | 2171.70 | 2610.83 | 2937.17 | 0.195 | 0.823 | 0.989 |
| Firmicutes | 70229.50 | 17138.75 | 69177.04 | 20340.41 | 69458.27 | 15828.92 | 0.046 | 0.955 | 0.989 |
| Verrucomicrobiota | 1360.96 | 2029.93 | 1324.35 | 2433.39 | 1398.48 | 2787.95 | 0.011 | 0.989 | 0.989 |

**Supplementary Table S2: Gut microbiome abundance in control, MCI and SCI at the genus level.** P-value generated by one-way ANOVA, with false discovery rate (FDR) correction for multiple testing.

| **Genus** | **Control**  **(n=50)** | | **SCI**  **(n=50)** | | **MCI**  **(n=50)** | | **F value** | **P-value** | **FDR** |
| --- | --- | --- | --- | --- | --- | --- | --- | --- | --- |
|  | **Mean** | **SD** | **Mean** | **SD** | **Mean** | **SD** |  |  |  |
| *Holdemania* | 1.12 | 1.14 | 3.58 | 3.24 | 1.10 | 1.14 | 20.140 | <0.001 | 0.007 |
| *UCG_009* | 1.13 | 1.41 | 1.04 | 1.23 | 0.27 | 0.51 | 11.400 | <0.001 | 0.007 |
| *Lachnoclostridium* | 23.83 | 16.15 | 43.29 | 30.72 | 29.44 | 17.06 | 9.446 | 0.001 | 0.044 |
| *Turicibacter* | 60.27 | 79.99 | 24.07 | 35.95 | 29.93 | 40.03 | 5.130 | 0.007 | 0.189 |
| *Lachnospiraceae_ND3007_group* | 116.70 | 92.88 | 132.60 | 96.04 | 78.22 | 73.04 | 4.597 | 0.012 | 0.255 |
| *Lactonifactor* | 0.14 | 0.40 | 0.58 | 1.13 | 0.31 | 0.55 | 4.253 | 0.016 | 0.300 |
| *Ruminococcus_gnavus_group* | 0.11 | 0.31 | 0.83 | 2.08 | 0.43 | 0.90 | 3.755 | 0.026 | 0.307 |
| *Bacteroides* | 1145.70 | 831.52 | 1678.54 | 1153.60 | 1471.77 | 969.21 | 3.606 | 0.030 | 0.324 |
| *Romboutsia* | 421.18 | 369.06 | 246.42 | 312.89 | 290.79 | 355.15 | 3.377 | 0.037 | 0.372 |
| *Intestinibacter* | 308.84 | 398.92 | 149.71 | 172.09 | 219.98 | 338.39 | 3.062 | 0.049 | 0.448 |
| *Fusicatenibacter* | 235.68 | 161.93 | 329.79 | 262.31 | 247.10 | 186.98 | 2.966 | 0.055 | 0.448 |
| *Lachnospiraceae_UCG_004* | 2.96 | 4.10 | 5.00 | 6.94 | 2.81 | 3.20 | 2.890 | 0.059 | 0.453 |
| *DTU089* | 2.78 | 3.86 | 1.40 | 1.94 | 1.79 | 2.78 | 2.816 | 0.063 | 0.460 |
| *Parasutterella* | 5.92 | 15.65 | 13.54 | 33.01 | 4.06 | 9.10 | 2.588 | 0.079 | 0.500 |
| *unidentified_Ruminococcaceae* | 5.06 | 11.68 | 10.06 | 22.47 | 3.48 | 5.01 | 2.571 | 0.080 | 0.500 |
| *Parabacteroides* | 124.64 | 115.60 | 222.08 | 331.41 | 161.08 | 128.52 | 2.566 | 0.080 | 0.500 |
| *Bifidobacterium* | 819.82 | 1362.41 | 416.54 | 483.27 | 527.65 | 645.06 | 2.514 | 0.085 | 0.500 |
| *Shuttleworthia* | 5.38 | 11.37 | 2.88 | 3.81 | 2.38 | 3.22 | 2.435 | 0.091 | 0.500 |
| *Coprobacter* | 6.24 | 11.97 | 10.00 | 14.11 | 5.00 | 7.73 | 2.432 | 0.092 | 0.500 |
| *Defluviitaleaceae_UCG_011* | 3.02 | 3.74 | 1.77 | 2.89 | 1.85 | 2.90 | 2.334 | 0.101 | 0.513 |
| *Oscillospira* | 0.82 | 1.62 | 0.40 | 0.71 | 0.42 | 0.71 | 2.284 | 0.106 | 0.513 |
| *Monoglobus* | 39.80 | 37.65 | 57.69 | 68.53 | 38.54 | 34.68 | 2.283 | 0.106 | 0.513 |
| *Family_XIII_UCG_001* | 7.94 | 6.94 | 5.60 | 4.49 | 6.35 | 5.00 | 2.231 | 0.111 | 0.520 |
| *Erysipelatoclostridium* | 11.36 | 21.75 | 22.79 | 44.76 | 12.13 | 15.61 | 2.194 | 0.115 | 0.520 |
| *Butyricimonas* | 6.58 | 10.63 | 7.13 | 12.82 | 14.29 | 31.71 | 2.118 | 0.124 | 0.541 |
| *Roseburia* | 105.44 | 104.08 | 162.33 | 178.58 | 132.94 | 136.81 | 1.948 | 0.146 | 0.578 |
| *Negativibacillus* | 6.14 | 9.62 | 8.90 | 14.42 | 12.02 | 19.69 | 1.862 | 0.159 | 0.578 |
| *Butyricicoccus* | 50.02 | 45.21 | 65.42 | 62.85 | 47.90 | 33.85 | 1.856 | 0.160 | 0.578 |
| *Dorea* | 246.36 | 143.07 | 235.52 | 115.79 | 290.96 | 183.05 | 1.853 | 0.161 | 0.578 |
| *Ruminococcus_torques_group* | 65.32 | 86.53 | 79.60 | 69.12 | 100.90 | 115.25 | 1.842 | 0.162 | 0.578 |
| *Desulfovibrio* | 20.96 | 35.43 | 8.81 | 23.60 | 19.42 | 40.57 | 1.836 | 0.163 | 0.578 |
| *Streptococcus* | 51.30 | 85.73 | 123.77 | 320.59 | 156.48 | 365.15 | 1.779 | 0.173 | 0.578 |
| *Eubacterium_ventriosum_group* | 65.46 | 151.98 | 32.52 | 25.73 | 38.17 | 38.58 | 1.774 | 0.173 | 0.578 |
| *Hungatella* | 0.80 | 1.62 | 2.81 | 7.15 | 7.23 | 29.21 | 1.768 | 0.174 | 0.578 |
| *Catenibacillus* | 0.74 | 1.50 | 0.46 | 1.38 | 0.27 | 0.71 | 1.756 | 0.176 | 0.578 |
| *Lacticaseibacillus* | 0.90 | 3.85 | 0.58 | 1.65 | 3.10 | 11.98 | 1.709 | 0.185 | 0.590 |
| *unidentified_Gastranaerophilales* | 0.54 | 1.68 | 0.69 | 1.85 | 1.48 | 4.13 | 1.610 | 0.203 | 0.620 |
| *Lachnospira* | 47.66 | 56.85 | 63.13 | 109.70 | 36.17 | 34.73 | 1.609 | 0.204 | 0.620 |
| *Faecalibacterium* | 704.26 | 513.26 | 895.96 | 581.74 | 785.65 | 539.99 | 1.522 | 0.222 | 0.648 |
| *Faecalitalea* | 4.08 | 10.48 | 9.33 | 22.59 | 4.31 | 15.14 | 1.518 | 0.223 | 0.648 |
| *UBA1819* | 4.00 | 5.35 | 3.10 | 3.92 | 5.90 | 12.52 | 1.470 | 0.233 | 0.664 |
| *Escherichia_Shigella* | 277.36 | 852.95 | 104.96 | 306.82 | 369.40 | 1018.23 | 1.394 | 0.251 | 0.681 |
| *Gordonibacter* | 1.82 | 2.63 | 0.98 | 1.72 | 1.52 | 3.05 | 1.386 | 0.253 | 0.681 |
| *Phocea* | 0.30 | 0.74 | 0.48 | 1.03 | 0.21 | 0.62 | 1.381 | 0.255 | 0.681 |
| *Adlercreutzia* | 9.76 | 10.62 | 11.10 | 12.54 | 14.70 | 21.16 | 1.328 | 0.268 | 0.691 |
| *Eubacterium_eligens_group* | 27.82 | 32.88 | 42.21 | 43.61 | 31.94 | 55.84 | 1.323 | 0.270 | 0.691 |
| *unidentified_Erysipelotrichaceae* | 9.76 | 17.12 | 8.46 | 37.92 | 2.38 | 6.08 | 1.289 | 0.279 | 0.691 |
| *Lachnospiraceae_FCS020_group* | 36.82 | 22.63 | 30.40 | 22.23 | 31.35 | 19.69 | 1.272 | 0.283 | 0.691 |
| *Sutterella* | 11.82 | 18.64 | 21.44 | 50.48 | 12.75 | 19.18 | 1.265 | 0.285 | 0.691 |
| *Odoribacter* | 12.12 | 12.45 | 14.94 | 15.44 | 11.04 | 9.08 | 1.227 | 0.296 | 0.691 |
| *Blautia* | 1175.92 | 587.50 | 1407.38 | 755.54 | 1260.94 | 865.82 | 1.211 | 0.301 | 0.691 |
| *Frisingicoccus* | 0.98 | 3.99 | 3.29 | 8.85 | 5.54 | 23.41 | 1.206 | 0.302 | 0.691 |
| *GCA_900066575* | 4.06 | 4.14 | 5.31 | 5.40 | 4.00 | 4.53 | 1.194 | 0.306 | 0.691 |
| *UC5_1_2E3* | 0.20 | 0.45 | 0.50 | 1.34 | 0.42 | 1.03 | 1.175 | 0.312 | 0.692 |
| *Veillonella* | 5.22 | 17.90 | 9.65 | 31.08 | 3.50 | 6.94 | 1.091 | 0.339 | 0.700 |
| *Prevotella_9* | 396.22 | 924.21 | 195.83 | 526.81 | 450.58 | 1122.81 | 1.085 | 0.341 | 0.700 |
| *Tyzzerella* | 7.16 | 19.74 | 4.71 | 13.10 | 11.04 | 28.67 | 1.064 | 0.348 | 0.700 |
| *Methanobrevibacter* | 336.18 | 669.38 | 211.06 | 374.14 | 361.23 | 537.90 | 1.058 | 0.350 | 0.700 |
| *UCG_003* | 16.74 | 28.81 | 15.29 | 21.65 | 10.40 | 15.59 | 1.036 | 0.357 | 0.700 |
| *Marvinbryantia* | 24.22 | 20.89 | 18.29 | 15.80 | 23.15 | 26.92 | 1.031 | 0.359 | 0.700 |
| *Haemophilus* | 4.36 | 12.82 | 32.75 | 201.64 | 2.56 | 9.50 | 1.029 | 0.360 | 0.700 |
| *Bilophila* | 8.08 | 9.68 | 10.90 | 10.10 | 10.50 | 11.94 | 1.018 | 0.364 | 0.700 |
| *GCA_900066755* | 0.32 | 0.71 | 0.17 | 0.43 | 0.31 | 0.62 | 0.997 | 0.372 | 0.700 |
| *UCG_008* | 0.32 | 0.89 | 0.15 | 0.50 | 0.40 | 1.16 | 0.988 | 0.375 | 0.700 |
| *Parvibacter* | 0.62 | 2.63 | 1.17 | 3.03 | 1.42 | 3.00 | 0.978 | 0.379 | 0.700 |
| *Corynebacterium* | 0.20 | 0.53 | 0.13 | 0.39 | 0.29 | 0.77 | 0.973 | 0.381 | 0.700 |
| *UCG_005* | 94.14 | 83.08 | 80.00 | 81.08 | 72.69 | 68.88 | 0.961 | 0.385 | 0.700 |
| *Eggerthella* | 3.46 | 14.11 | 7.81 | 21.48 | 5.31 | 10.27 | 0.916 | 0.402 | 0.722 |
| *Ruminococcus* | 628.98 | 491.31 | 613.60 | 545.71 | 506.04 | 474.47 | 0.854 | 0.428 | 0.753 |
| *Christensenella* | 0.26 | 0.75 | 0.52 | 1.40 | 0.38 | 0.70 | 0.837 | 0.435 | 0.753 |
| *Lachnospiraceae_NK4A136_group* | 76.24 | 92.21 | 106.42 | 95.02 | 97.50 | 161.80 | 0.816 | 0.444 | 0.753 |
| *unidentified_Lachnospiraceae* | 0.78 | 1.27 | 1.10 | 2.30 | 1.29 | 2.32 | 0.811 | 0.447 | 0.753 |
| *Actinomyces* | 2.56 | 2.82 | 3.63 | 5.63 | 2.98 | 3.62 | 0.806 | 0.448 | 0.753 |
| *Holdemanella* | 126.22 | 224.72 | 214.31 | 560.79 | 208.42 | 368.27 | 0.724 | 0.487 | 0.807 |
| *Anaerofustis* | 0.38 | 0.75 | 0.29 | 0.62 | 0.23 | 0.52 | 0.693 | 0.502 | 0.810 |
| *Merdibacter* | 0.94 | 1.80 | 1.04 | 4.28 | 0.42 | 1.46 | 0.693 | 0.502 | 0.810 |
| *Enterobacter* | 49.44 | 345.85 | 89.06 | 419.64 | 13.52 | 68.22 | 0.683 | 0.507 | 0.810 |

SD= standard deviation

| **Supplementary Table S3: Serum metabolite concentrations in control, subjective cognitive impairment (SCI) and mild cognitive impairment (MCI) by LC-MS/MS.** Mean ±SD. P-value generated from one-way ANOVA. <LOD= below the limit of detection. | | | | | |
| --- | --- | --- | --- | --- | --- |
| **Pathway** | **Metabolite** | **Control**  **(µM)** | **SCI**  **(µM)** | **MCI**  **(µM)** | **P-value** |
| TMAO  Pathway | Trimethylamine | 0.94 ± 0.31 | 0.96 ± 0.78 | 1.03 ±1.15 | 0.86 |
|  | Trimethylamine N-oxide | 4.49 ± 2.96 | 5.81± 8.46 | 7.04 ± 9.63 | 0.25 |
|  | Choline | 23.25 ± 6.20 | 19.79 ± 4.93 | 20.09 ± 4.81 | **<0.01** |
| Tryptophan Pathway | Tryptophan | 36.09 ± 4.78 | 36.62 ± 4.44 | 36.08 ± 6.96 | 0.79 |
|  | Kynurenine | 1.07 ± 0.27 | 1.16 ± 0.29 | 1.16 ± 0.27 | 0.19 |
|  | Serotonin | 0.35 ± 0.16 | 0.31 ± 0.17 | 0.35 ± 0.14 | 0.32 |
|  | 5-hydroxyindole acetic acid | 0.05 ± 0.02 | 0.04 ± 0.01 | 0.03 ± 0.01 | **<0.01** |
|  | Kynurenic acid | 0.03 ± 0.01 | 0.04 ± 0.01 | 0.04 ± 0.01 | 0.18 |
|  | Xanthurenic acid | 0.01 ± 0.01 | 0.02 ± 0.01 | 0.02 ± 0.01 | 0.88 |
|  | 3-hydroxyanthranilic acid | <LOD | <LOD | <LOD | - |
|  | Anthranilic acid | 0.07 ± 0.06 | 0.09 ± 0.07 | 0.06 ± 0.06 | 0.11 |
|  | Indole | <LOD | <LOD | <LOD | - |
|  | Indole-3 -acetic acid | 1.33 ± 0.48 | 1.40 ± 0.48 | 1.36 ± 0.68 | 0.84 |
|  | Indole-3- lactic acid | 0.54 ± 0.23 | 0.49 ± 0.14 | 0.50 ± 0.13 | 0.42 |
|  | Indole-3- carboxaldehyde | 0.03 ± 0.01 | 0.03 ± 0.01 | 0.03 ± 0.01 | 0.50 |
|  | Indole-3 -propionic acid | 1.34 ± 0.67 | 1.19 ± 0.62 | 0.96 ± 0.68 | **0.02** |
|  | Methyl indole 3- acetate | 0.03 ± 0.02 | 0.03 ± 0.02 | 0.03 ± 0.02 | 0.41 |
|  | Indoxyl sulfate | 2.41 ± 1.24 | 3.54 ± 2.03 | 3.18 ± 1.55 | **<0.01** |
| *P*-Cresol  Pathway | *P*-cresol sulfate | 19.93 ± 10.87 | 22.79 ± 13.22 | 23.45 ± 12.21 | 0.31 |
|  | *P*-cresol glucuronide | 0.13 ± 0.14 | 0.13 ± 0.20 | 0.12 ± 0.12 | 0.87 |
| Bile Acid  Pathway | CA | 0.23 ± 0.35 | 0.26 ± 0.48 | 0.24 ± 0.40 | 0.95 |
|  | CDCA | 0.01 ± 0.01 | 0.01 ± 0.02 | 0.01 ± 0.02 | 0.71 |
|  | HDCA | 0.02 ± 0.02 | 0.02 ± 0.02 | 0.03 ± 0.06 | 0.17 |
|  | GCDCA | 0.39 ± 0.26 | 0.49 ± 0.90 | 0.41 ± 0.33 | 0.64 |
|  | GDCA | 0.16 ± 0.15 | 0.20 ± 0.33 | 0.23 ± 0.24 | 0.46 |
|  | GCA | 0.10 ± 0.08 | 0.23 ± 0.84 | 0.14 ± 0.19 | 0.43 |
|  | DCA | 0.26 ± 0.26 | 0.27 ± 0.31 | 0.31 ± 0.45 | 0.75 |
|  | TCDCA | 0.05 ± 0.05 | 0.10 ± 0.35 | 0.06 ± 0.07 | 0.43 |
|  | TDCA | 0.02 ± 0.02 | 0.04 ± 0.10 | 0.05 ± 0.07 | 0.21 |
|  | GUCDA | 0.06 ± 0.08 | 0.05 ± 0.07 | 0.05 ± 0.06 | 0.75 |
|  | TUDCA | <LOD | <LOD | <LOD | - |
|  | TLCA | <LOD | <LOD | <LOD | - |
|  | THCA | <LOD | <LOD | <LOD | - |
|  | GLCA | 0.02 ± 0.02 | 0.01 ± 0.01 | 0.03 ± 0.06 | 0.19 |
|  | GHCA | <LOD | <LOD | <LOD | - |
|  | GHDCA | <LOD | <LOD | <LOD | - |
|  | UDCA | 0.03 ± 0.05 | 0.04 ± 0.07 | 0.03 ± 0.06 | 0.83 |
|  | TCA | 0.03 ± 0.03 | 0.09 ± 0.40 | 0.04 ± 0.06 | 0.41 |
|  | HCA | 0.01 ± 0.01 | 0.01 ± 0.01 | 0.01 ± 0.1 | 0.39 |
|  | LCA | 0.03 ± 0.03 | 0.04 ± 0.03 | 0.05 ± 0.07 | 0.13 |

**Supplementary Table S5: Comparison of area under the receiver operator curve (AUC)**

| Machine Learning Model | Classification | | |
| --- | --- | --- | --- |
|  | Control vs. SCI vs. MCI  (32 metabolites)  AUC | Control vs. SCI vs. MCI  (6 metabolites)  AUC | Control vs. MCI  (6 metabolites)  AUC |
| Random Forest | 0.65 | 0.79 | 0.84 |
| AdaBoost | 0.58 | 0.68 | 0.87 |
| Naïve Bayes | 0.63 | 0.72 | 0.90 |

**Supplementary Table S6: Summary of diagnostic performance obtained from metabolite and microbiome genera Random Forest models.**

| **RF Model** | **Precision** | **Recall** | **F1 Score** |
| --- | --- | --- | --- |
| Control vs. SCI vs. MCI  (6 metabolites) | 0.71 ± 0.05 | 0.68 ± 0.06 | 0.67± 0.05 |
| Control vs. SCI vs. MCI  (11 genera) | 0.44 ± 0.04 | 0.43 ± 0.04 | 0.43 ± 0.04 |

Mean ±SD

RF= Random Forest

**References**

1. Blokker, B. A. *et al.* Fine‐Tuning of Sirtuin 1 Expression Is Essential to Protect the Liver From Cholestatic Liver Disease. *Hepatology* **69**, 699–716 (2019).

2. Anesi, A. *et al.* Metabolic Profiling of Human Plasma and Urine, Targeting Tryptophan, Tyrosine and Branched Chain Amino Acid Pathways. *Metabolites* **9**, E261 (2019).

3. Hoyles, L. *et al.* Metabolic retroconversion of trimethylamine N-oxide and the gut microbiota. *Microbiome* **6**, 73 (2018).
